# Prescribing pattern of anti-hypertensive medications among hypertensive outpatients at selected hospitals of South Gondar Zone, Amhara, Ethiopia: a hospital based cross sectional study

**DOI:** 10.1186/s40360-022-00635-w

**Published:** 2022-12-30

**Authors:** Taklo Simeneh Yazie, Yohannes Shumet Yimer, Abebe Muche Belete, Getaye Tessema Desta

**Affiliations:** 1 Pharmacology and Toxicology Unit, Department of Pharmacy, College of Health Sciences, Debre Tabor University, Debre Tabor, Amhara, Ethiopia; 2 Department of Pharmacy, College of Health Sciences, Debre Tabor University, Debre Tabor, Amhara, Ethiopia; 3 Department of Medicine, College of Medicine and Health Sciences, Debre Berhan University, Debre Berhan, Amhara, Ethiopia

**Keywords:** Prescription pattern, Blood pressure control, Drug-drug interaction, Factors

## Abstract

**Background:**

Irrational prescription has a lion share for uncontrolled blood pressure. There is no study assessing prescription pattern among hypertensive patients at the study sites. Therefore, the objective of the current study was to evaluate prescription patterns for hypertension and blood pressure (BP) control at randomly selected hospitals of South Gondar Zone.

**Methods:**

A hospital based cross sectional study was conducted from December 1, 2020 to February 30, 2021. Hypertensive patients were selected by systematic random sampling proportionally from study hospitals. Structured questionnaires were used to collect socio-demographic chacteristics and adherence. Data abstraction form was used to collect prescription patterns, BP level and other necessary information. The association of prescription patterns and other variables with blood pressure control was determined by using binary logistic regression.

**Results:**

All recruited 423 patients were included in data analysis. Among prescriptions for hypertension, on average 93.5% were found to be in line with WHO guideline. About 53% of prescriptions for hypertension were monotherapies. Patient level low medication regimen complexity, and monotherapy were associated with blood pressure control (Ajusted Odds Ratio [AOR] = 2.04, [1.07–3.91]; AOR = 3.83 [1.42–10.35], respectively). Patients with inappropriate drug selection, and non-adherence were less likely to have controlled BP (AOR = 0.47 [0.26–0.85]; AOR = 0.52 [0.34–0.85], respectively). Moreover, patients who didn’t have health insurance and follow regular aerobic exercise were less likely to have controlled BP (AOR = 0.42 [0.26–0.68]; AOR = 0.53 [0.32–0.88], respectively).

**Conclusion:**

Diuretics were the most frequently prescribed drug in monotherapy and in combination with calcium channel blockers (CCBs) as dual therapy. On average, more than 90% of prescription was in accordance with WHO guideline and around one-third of participants experienced at least one moderate or major drug-drug interaction. Patient level low medication regimen complexity and monotherapy were positively associated with BP control whereas, non-adherence, inappropriate drug selection, having no health insurance, and didn’t follow regular aerobic exercise were negatively associated with BP control. Clinicians should be adherent to treatment guidelines and focus on modifiable factors to improve BP control.

## Background

Hypertension can be defined as average systolic and/or average diastolic blood pressure levels equal and greater than 140 mmHg and 90 mmHg, respectively or reported use of antihypertensive drugs. Hypertension is a major public health problem that considerably increases the risk of heart, brain, kidney and other disease. Worldwide, around 1.4 billion people were estimated to have high blood pressure [1, 2]. Hypertension is a leading cause of premature death worldwide and its prevalence was higher in low-income and middle-income countries (LMICs) than developed countries [3, 4]. In 2015, 8.5 million deaths were estimated and attributable to systolic blood pressure > 115 mmHg, 88% of which were in low-income and middle-income countries [4].

Antihypertensive drugs are known to have clear benefits in reducing the rate of cardiovascular complications and risk of adverse health problems [5]. The use of treatment guidelines enhances the proper use of medications but there are concerns about adherence rate to treatment guidelines across countries [6–9]. Despite the existence of cost-effective treatment options, only around 14% of hypertensive patients have their blood pressure under control across the world [1, 2]. The level of blood pressure control is lower in LMICs compared to developed countries (1). In Ethiopia, a systematic review showed that around 48% of hypertensive patients under treatment had uncontrolled blood pressure [10]. Among several factors, inappropriate prescription may have the lion share for the outcome of poor blood pressure control [11, 12]. Irrational prescription of medications has been confirmed to be associated with higher hospital admission rate [13, 14]. Furthermore, its association with mortality was evidenced by a longitudinal study [13], and a systematic review [15].

Current Ethiopian treatment guideline recommends thiazide and thiazide like diuretics (TLDs), calcium channel blockers (CCBs), angiotensin convertase enzyme inhibitors (ACEIs) or Angiotensin II type 1 receptor blockers (ARBs) as preferred first line agents for patients with hypertension, which is in line with current World Health Organization (WHO) and Joint National Committee-Eight (JNC-8) recommendations. The treatment can be stepped up further in terms of dose and combination of drugs from different classes if blood pressure is not under control [2, 16, 17].

To date, there is paucity of data regarding prescription pattern of antihypertensive drugs in Ethiopia. This is a major concern considering hypertension is a big public health problem in Ethiopia and consequence of poor blood pressure control [10, 12]. As irrational prescription is one of a contributing factor for poor blood pressure control and there is no data concerning it in the study sites, we aimed to assess prescription pattern of antihypertensive drugs among hypertensive outpatients at selected hospitals of South Gondar Zone.

## Methods

### Study area

The study areas were four randomly selected hospitals which are found in South Gondar Zone, Amhara Regional State, Ethiopia. Eleven administrative zones are found in this Zone and the health services have been provided with 1 Comprehensive Specialized Hospital, 7 primary hospitals and 95 health centers in this zone. Above 2 million people was living in this zone according to the 2007 national census conducted by Central Statistical Agency (CSA) of Ethiopia [18].

### Study design and period

The study design was a hospital base cross sectional study which was conducted from December 1, 2020 to February 30, 2021.

### Source and study population

The source population was all hypertensive patients who have follow-up and attending the selected hospitals. Patients who fulfilled the inclusion criteria and receiving anti-hypertensive drugs were considered as the study population.

### Sample size and sampling technique

The single population proportion formula was employed to determine the sample size as shown below:

$$\mathrm n=\frac{\mathrm z^2\ast\mathrm P\ast(1-\mathrm P)}{\mathrm w^2}$$

**Fig. 1:**
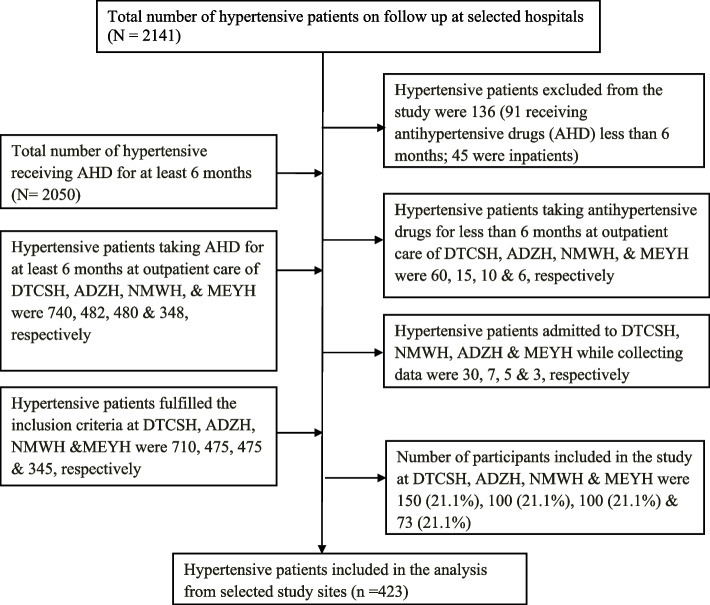
Sampling procedure of the study used in analysis at selected hospitals of South Gondar Zone, Amhara, Ethiopia. Abbreviations: DTCSH, Debre Tabor Comprehensive Specialized Hospital; ADZH, Addis Zemen Hospital; NMWH, Nifas Mewcha Hospital; MEYH, Mekane Eyesus Hospital

Where n is the required sample size for the population, Z is the standard normal distribution set as 1.96, P is prevalence of outcome variable estimated to determine the sample size, and W is the degree of accuracy which is 0.05. There is no previous study regarding prescription pattern of anti-hypertensive drugs in similar study areas to show clear magnitude of the problem, so a *p* value of 0.5 was used. Then, the sample size was determined as, *n*= (1.96)^2^ *0.5*((1–0.5)/(0.05)^2^ = 384. After adding 10% for non-responding rate, the final sample size was found to be 423. Debre Tabor Comprehensive Specialized Hospital was selected purposively as it is the only Comprehensive Specialized Hospital of this zone. Random was employed to select 3 primary hospitals which were Addis Zemen, Mekane Eyesus, Nifas Mewcha. Then, 423 study participants were interviewed and their charts were reviewed by employing systematic random sampling and proportional allocation to study hospitals (Fig. 1). Assuming that all study population come to each respective study sites from December 1, 2020 to February 30, 2021, systematic random sampling was used. To determine sampling interval, the total population of study sites was divided by the sample size of study sites. Lastly, the sampling interval was 4, and every 4th of study participants was included till the chosen sample was attained.

### Study variables

➢ Dependent variables: Prescribing pattern of antihypertensive medications, and blood pressure control
➢ Independent variables: age, sex, co-morbidity, duration of hypertension treatment, adherence to medication, prescription pattern of antihypertensive medications, class of antihypertensive drug, monotherapy, combination therapy, dose frequency, education level, income level, marital status, drug-drug interactions

### Inclusion and exclusion criteria

The inclusion criteria were adult hypertensive patients who receive antihypertensive drug for at least 6 months and willing to take part in the study. Patients with incomplete medical records, inpatients, cognitive and hearing impairment were excluded from the study.

### Operational definitions

Controlled hypertension was operationally defined according to JNC-8 and WHO guideline for hypertension. These two guidelines are different in target BP cut off points. According to JNC-8, controlled hypertension was considered when BP < 150/90 mmHg in hypertensive patients aged 60 or older, or BP < 140/90 mmHg in hypertensive patients aged less than 60 years and all ages of hypertensive patients with diabetes mellitus or non-diabetic chronic kidney disease [17], whereas according to WHO guideline controlled hypertension was considered when BP < 140/90 for hypertension with no comorbidity, or BP < 130/90 mmHg for hypertension with diabetes mellitus, chronic kidney disease and known cardiovascular diseases [2].

Uncontrolled hypertension was considered according to JNC-8 when BP ≥ 150/90 mmHg in hypertensive patients aged 60 or older, or BP ≥ 140/90 mmHg in hypertensive patients aged less than 60 years and all ages of hypertensive patients with diabetes mellitus or non-diabetic chronic kidney disease [17], whereas according to WHO guideline, uncontrolled hypertension was considered when BP ≥ 140/90 mm Hg for hypertension with no comorbidity, or BP ≥ 130/90 mmHg for hypertension with diabetes mellitus, chronic kidney disease and known cardiovascular diseases [2].

Adherence was assessed by using eight item medication adherence scale (MAS-8) scale and those patients with MAS score of 8 was considered as adherent to their medication [19].

Regular aerobic exercise was considered for patients engaged in moderate aerobic exercise such as walking briskly, recreational swimming, riding bicycle, and tennis for at least 150 min per week (30 min per day for 5 days per week) [20].

Adherence of prescribers to guideline was assessed based on the recommendation of WHO guideline for hypertension management, and adherence to guideline was considered when prescribers follow 100% of the guideline recommendations [2].

### Data collection procedures

Socio-demographic, Clinical data, and patients’ details of current medications were gathered from the chart. Socioeconomics, adherence to medication, and other required data that were not found from the chart were collected by interviewing the patients using structured questionnaire. The questionnaire was translated to Amharic language and back to English in order to maintain its consistency.

Adherence to medication was evaluated by using an eight item medication adherence scale (MAS-8). For the first seven items, the value for “Yes” = 0 and “No” = 1 was given whereas for item number five, the values for “Yes” and “No” are reversed and for the last item a five-point likert response are used with options “never”, “once in a while”, “sometimes”, “usually”, and “always” [19]. A recently measured BP value of participants was used to evaluate whether BP is controlled or not. At selected hospitals, BP was measured according to the recommendations from clinical practice guideline of American College of Cardiology/American Heart Association Task Force. Two BP reading of two minute apart of a visit were taken and the average was considered to determine the status of BP [21].

Drug-drug interaction was checked by using IBM Micromedex® online drug-drug interaction checker which is accessible at http://www.micromedexsolutions.com [22].

### Data quality control

Data quality control was ensured by conducted pretest on 5% of the sample size before the start of actual data collection at Ebinat primary hospital, and providing training for four pharmacists as data collectors who were assigned one for each study site to interview the participant and to review the patient charts. Training was given for two days and issues addressed were objectives, data collection methods, and ethical concerns. The completeness and correctness of the filled questionnaires were checked daily by the principal investigator.

### Data processing and analysis

SPSS version 21.0 was used to enter and analyze data. Frequencies were used to describe categorical variables whereas variables with continuous scale were described by using means and standard deviation (SD). Binary logistic regression was employed to determine association of predictive and outcome variables. Variables with *p* value < 0.2 in univariate analysis were included in multivariate logistic regression and a statistical significance was considered at a *p* value < 0.05.

## Results

### Patient characteristics

A total of 423 participants were recruited and included in the final data analysis. The mean age of the respondents was 58.48 ± 12.96 years. Their mean BP was 132.3/82.1 mmHg and more than one-half of the study participants was female (59.8%). Around two-thirds of respondents were above 50 years of age (69.7%). Regarding to comorbidities, 57.9% of respondents have been living with other comorbidities, with diabetes mellitus (13%) ranking first followed by cardiovascular disease (11.8%) (Table 1).

**Table 1:** Baseline characteristics of respondents

**Variables**	**Variables category**	**Frequency n (%)**
Sex	Female	253 (59.8)
Age (years)	23–30	10 (2.4)
	31–40	33 (7.8)
	41–50	85 (20.1)
	51–60	127 (30)
	61–70	96 (22.7)
	70+	72 (17.0)
Residence	Urban	267 (63.1)
	Rural	156 (36.9)
Comorbidities	Diabetes mellitus	55 (13)
	CVDs	50 (11.8)
	Dyslipidemia	25 (5.9)
	Asthma	21 (5.0)
	Arthritis	19 (4.5)
	Others ^a^	76 (18)

*Abbreviations:CVDs* Cardiovascular diseases, *n* number of patients

^a^ Includes Dyspepsia, Hyperthyroidism, Pneumonia, Epilepsy, Typhoid fever, Chronic kidney disease etc

### Prescription patterns of antihypertensive medications

**Fig. 2:**
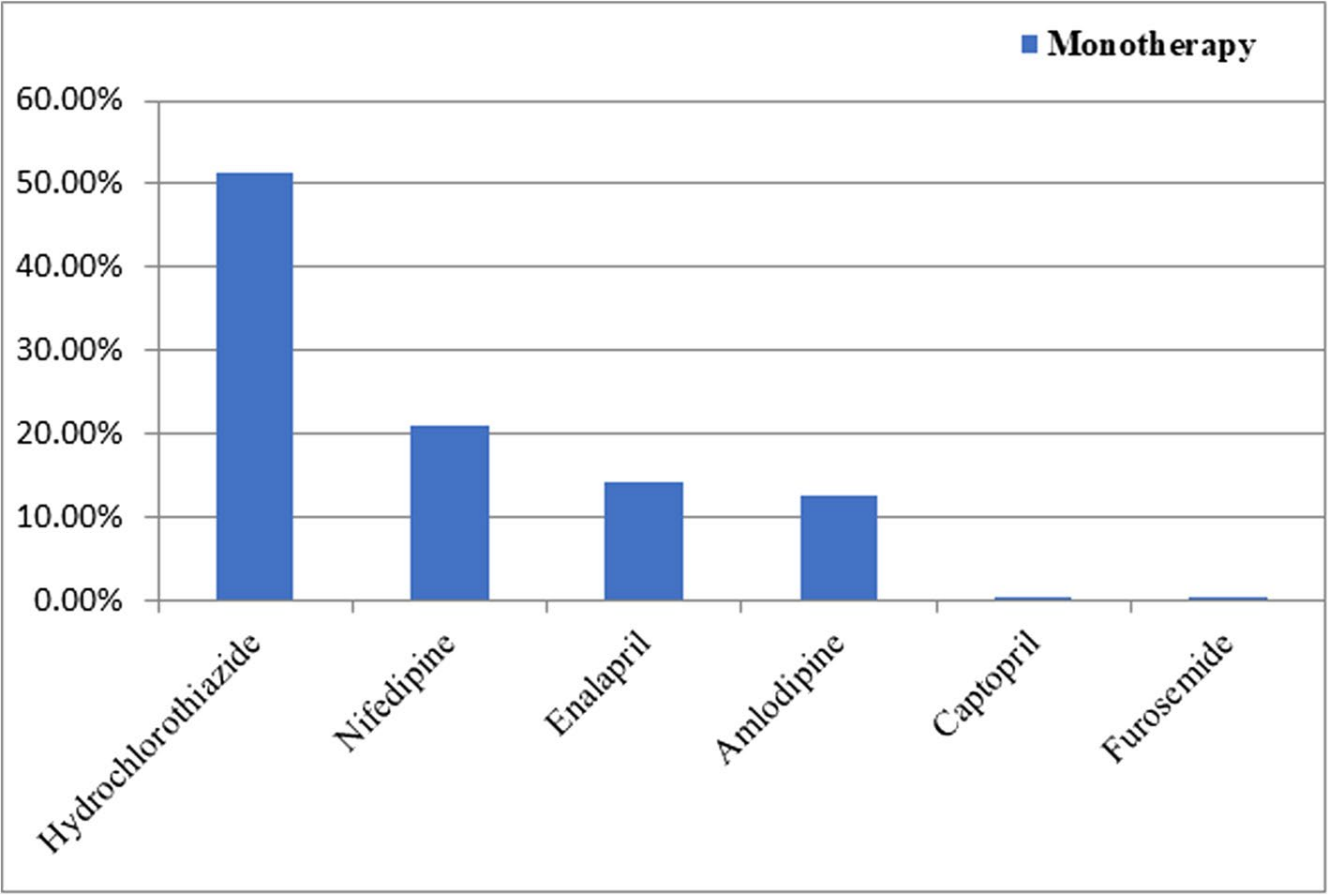
Types of antihypertensive monotherapy

Hydrochlorothiazide, nifedipine, enalapril, amlodipine, furosemide, and atenolol were among antihypertensive drugs prescribed. The most commonly prescribed antihypertensive monotherapy was hydrochlorothiazide (51.3%) followed by nifedipine (21%). Furosemide and captopril was the least single antihypertensive agent used (0.45%) (Fig. 2).

**Fig. 3:**
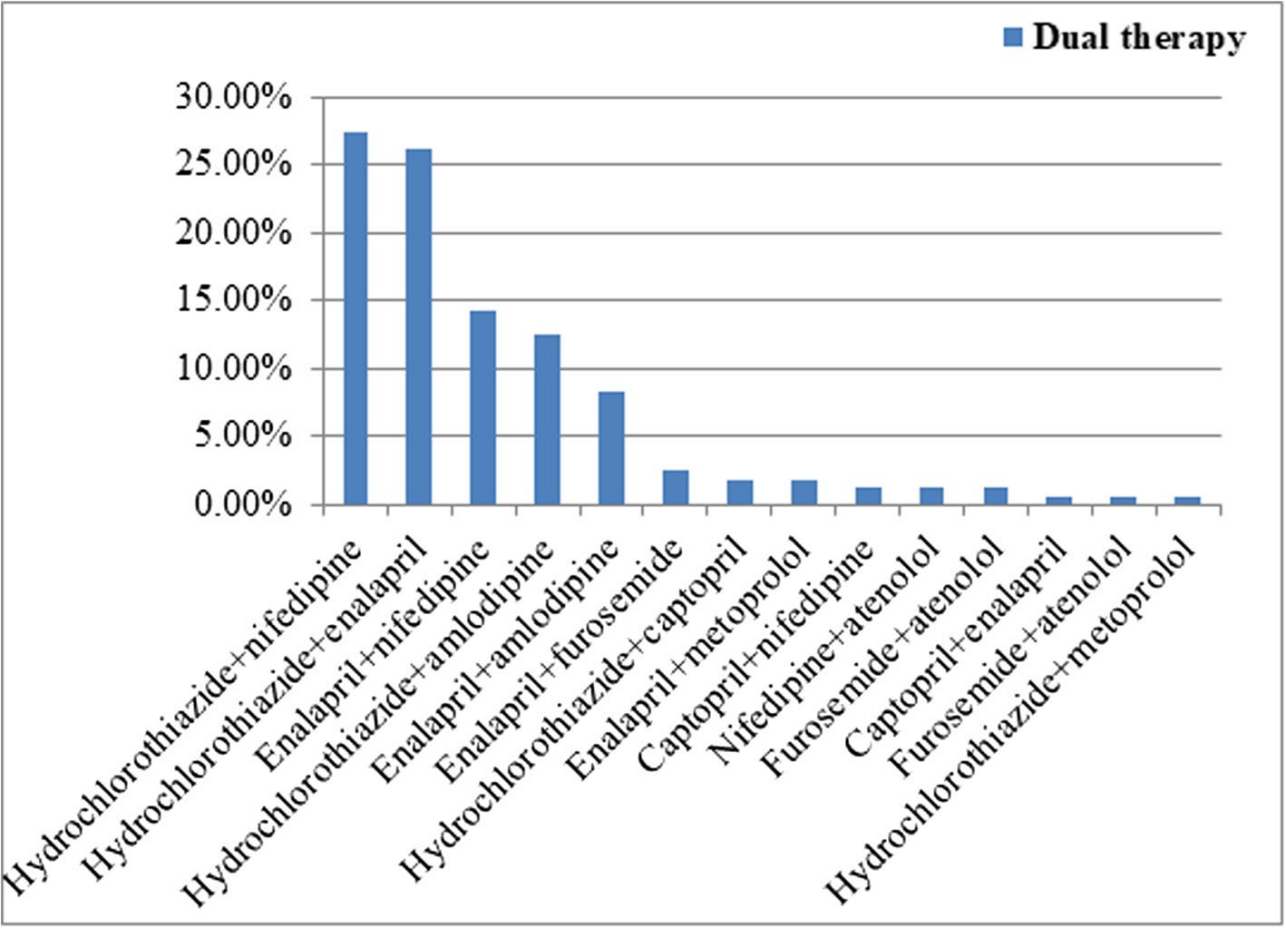
Types of dual antihypertensive therapies

Hydrochlorothiazide plus nifedipine was the most commonly prescribed dual therapy (27.5%) for hypertension in the present study. The second most commonly prescribed dual therapy was hydrochlorothiazide plus enalapril which accounts 26.3% of dual combination prescriptions. Furosemide plus atenolol was one of the less frequently prescribed dual therapies (Fig. 3).

**Fig. 4:**
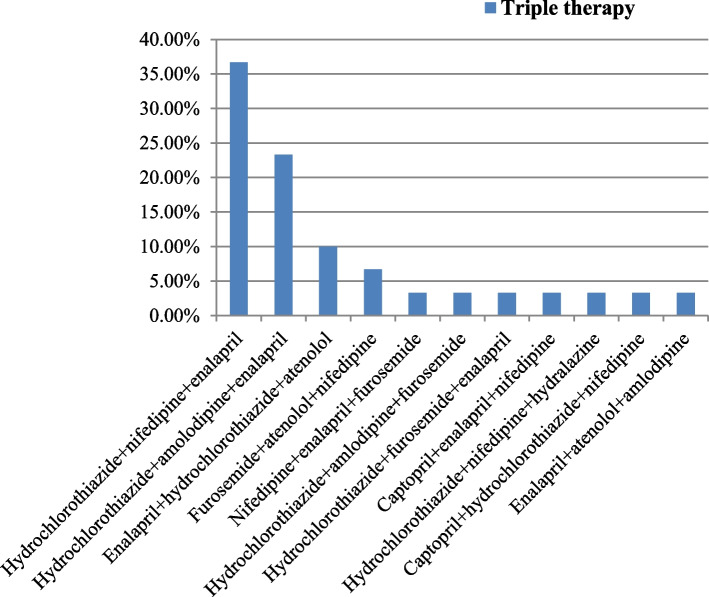
Types of triple antihypertensive therapies

Concerning to triple therapy, hydrochlorothiazide plus nifedipine plus enalapril was the most commonly prescribed triple antihypertensive combination therapy (36.7%). The second most commonly prescribed triple combination therapy was found to be hydrochlorothiazide plus amlodipine plus enalapril (23.3%). Atenolol plus enalapril plus amlodipine was one of the least commonly prescribed combinations among triple antihypertensive therapies (3.3%) (Fig. 4).

**Fig. 5:**
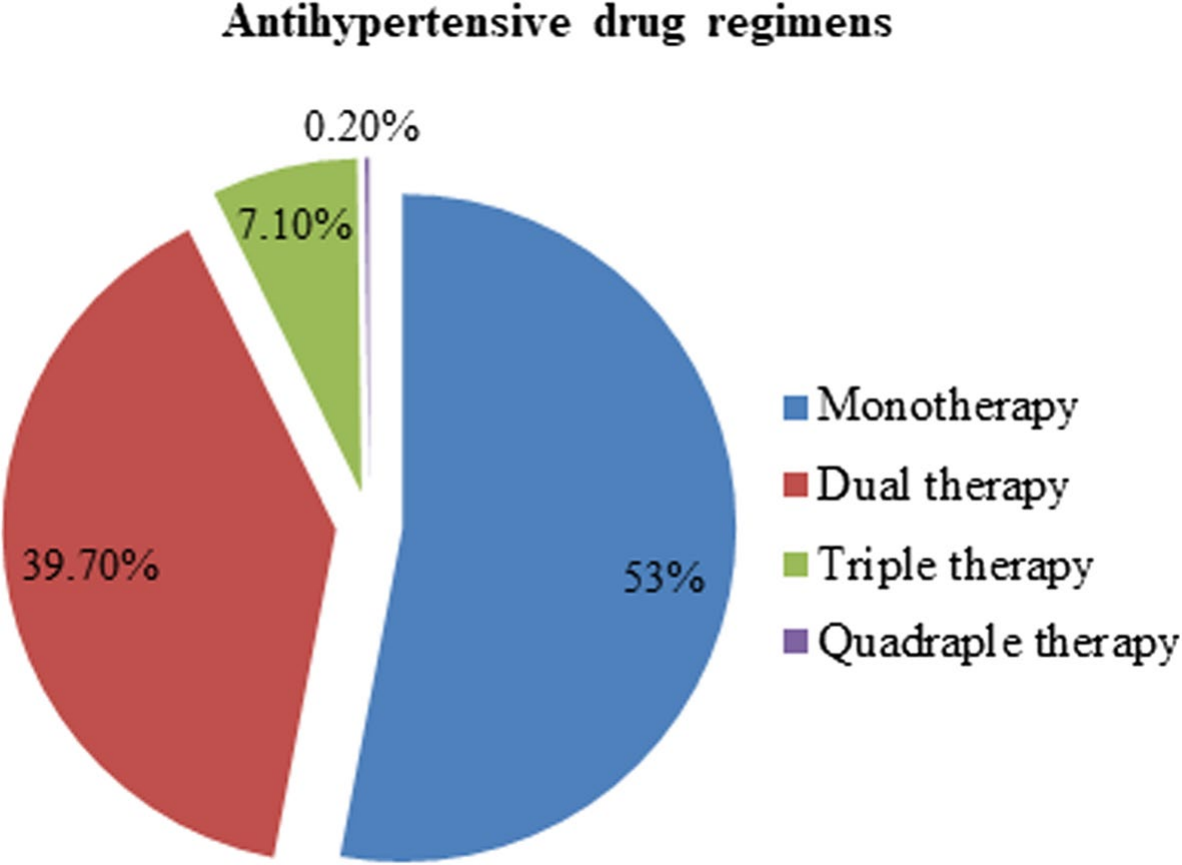
Antihypertensive drug regimen

Among antihypertensive drug regimens, monotherapy was the most frequently prescribed regimen (53%) whereas quadruple therapy was the least prescribed regimen (0.2%) (Fig. 5).

Among co-medications to hypertensive patients, paracetamol and metformin were the most commonly prescribed drugs, which account 12.7% and 10%, respectively (Table 2).

**Table 2:** Co-medications prescribed for hypertensive patients

**Drugs**	**Frequency, n (%)**	**Drugs**	**Frequency, n (%)**
**Antibiotics**		**Antiasmathics**	
Ciprofloxacillin	10 (2.1)	Salbutamol puff	17 (3.5)
Amoxicillin	9 (1.9)	Prednisolone	8 (1.7)
Azithromycin	6 (1.2)	Beclomethasone puff	2 (0.4)
Norfloxacillin	2 (0.4)		
Ceftriaxone	2 (0.4)		
Metronidazole	1 (0.2)		
Crystalline penicillin	1 (0.2)		
Cloxacillin	1 (0.2)		
Cotrimoxazole	1 (0.2)		
**Supplements**		**Statin**	
Folic acid	3 (0.6)	Atorvastatin	37 (7.7)
Calcium gluconate	1 (0.2)	Rosuvastatin	15 (3.1)
Neurobin	1 (0.2)		
**Beta blocker**		**Antiplatelet**	
Propranolol	5 (1.0)	Aspirin	29 (6.0)
Metoprolol	3 (0.6)		
Timolol eye drop	1 (0.2)		
**Anticonvulsant**		**Diuretics**	
Phenytoin	3 (0.6)	Furosemide	30 (6.2)
Phenobarbital	2 (0.4)	Spironolactone	14 (2.9)
Gabapentin	1 (0.2)		
Carbamazepine	1 (0.2)		
**Antidiabetics**		**Ant thyroid drugs**	
Metformin	48 (10.0)	propylt hiouracil	5 (1.0)
Glibenclamide	19 (3.9)		
Insulin	8 (1.7)		
**NSAIDS**		**Others**	
Indometacin	13 (2.7)	Paracetamol	61 (12.7)
Ibuprofen	6 (1.2)	Omeprazole	32 (6.6)
Diclofenac	6 (1.2)	Amitryptylline	21 (4.4)
Meloxicam	3 (0.6)	Medicinal plants	16 (3.3)
		Tramadol	16 (3.3)
		ART	5 (1.0)
		Albendazole	2 (0.4)
		Dexthrometorphan	2 (0.4)
		Benzylbenzonate	1(0.2)
		Resiperidone	1(0.2)
		Carbidopa-levodopa	1(0.2)

### Compliance of prescribing pattern to treatment guidelines

Prescribing pattern of prescribers was evaluated based on WHO pharmacologic treatment of hypertension, and JNC-8. Compliance of prescribing to WHO and JNC-8 guideline was high in this study, which accounts on average 93.5% and 95.2%, respectively. Concerning to dose frequency and dose, 100% of dose frequency and 98.3% of dose were found to be in accordance with WHO guideline recommendations. Regarding to drug selection, 82.3% of antihypertensive drug prescriptions was in accordance with WHO guideline recommendations. Among 17.7% inappropriate antihypertensive drug selections, 9.2% accounts for inappropriate drug selection for compelling indications. According to IBM Micromedex® drug-drug interaction checker, 34% of participants were experienced at least one moderate or one major or both drug-drug interactions. Among observed drug-drug interactions, 5.4% was a moderate plus major interaction (Table 3).

**Table 3:** Compliance of prescribing pattern to guideline recommendations, and degree of drug-drug interactions

**Variable**	**Compliance to guidelines n (%)**	
	**WHO**	**JNC-8**
**Drug selection**		
Effective drug	348 (82.3)	369 (87.2)
Ineffective drug	75 (17.7)	54 (12.8)
**Dose**		
Normal	342 (98.3)	363 (98.4)
Too low	4 (1.1)	4 (1.1)
Too high	2 (0.6)	2 (0.5)
**Dose frequency**		
Appropriate	348 (100.0)	369 (100.0)
Inappropriate	0 (0.0)	0 (0.0)
**Drug-drug interaction**		
No interaction	279 (66.0)	279 (66.0)
Moderate	103 (24.3)	103 (24.3)
Major	41 (9.7)	41 (9.7)

When we saw the prescription pattern of antihypertensive drugs, more inappropriate drug selection (34.67%) was found at Debre Tabor Comprehensive Specialized Hospital whereas few inappropriate drug selections (17.33%) was found at Nefas Mewcha Hospital. Regarding to drug-drug interactions, more drug-drug interaction (43.06%) was found at Debre Tabor Comprehensive Specialized Hospital and few (13.19%) was found at Mekane Eyesus Hospital (Table 4).

**Table 4:** Prescription pattern of medications by study hospitals

**Variable**	**Study hospitals, n (%)**				**Total, n (%)**
	**DTCSH**	**NMWH**	**ADZH**	**MEYH**	
**Hypertension specific regimen complexity**					
Low	67 (44.7)	59 (59)	65 (65)	45 (61.6)	236 (55.8)
Moderate	77 (51.3)	37 (37)	34 (34)	25 (34.3)	173 (40.9)
High	6 (4)	4 (4)	1 (1)	3 (4.1)	14 (3.3)
**Patient level regimen complexity**					
Low total	22 (14.7)	15 (15)	34 (34)	16 (21.9)	87 (20.5)
Moderate total	71 (47.3)	40 (40)	37 (37)	41 (56.2)	189 (44.7)
High total	57 (38)	45 (45)	29 (29)	16 (21.9)	147 (34.8)
**Medication adherence**					
Adherent	83 (55.3)	18 (18)	56 (56)	3 (4.1)	160 (37.8)
Non adherent	67 (44.7)	82 (82)	44 (44)	70 (95.9)	263 (62.2)
**Blood pressure control**					
Controlled	47 (31.3)	31 (31)	72 (72)	25 (34.3)	175 (41.37)
Uncontrolled	103(68.7)	69 (69)	28 (28)	48 (65.8)	248 (58.63)
**Number of antihypertensive drug**					
Monotherapy	67 (44.7)	58 (58)	57 (57)	42 (57.5)	224 (52.9)
Dual therapy	69 (46)	38 (38)	39 (39)	23 (31.5)	169 (40)
Three or more	14 (9.3)	4 (4)	4 (4)	8 (10)	30 (7.1)
**Drug selection**					
Effective	124 (82.7)	87 (87)	85 (85)	52 (71.2)	348 (82.3)
Ineffective	26 (17.3)	13 (13)	15 (15)	21(28.8)	75 (17.7)
**Drug-drug interaction**					
No interaction	88 (58.7)	67 (67)	70 (70)	54 (74)	279 (66)
Moderate	44 (29.3)	19 (19)	22 (22)	18 (24.7)	103 (24.3)
Major	18 (12)	14 (14)	8 (8)	1 (1.4)	41 (9.7)
**Dose**					
Normal	147 (98)	98 (98)	100 (100)	72 (98.6)	417 (98.6)
Too low	2 (1.3)	2 (2)	0 (0)	0 (0)	4 (0.9)
Too high	1 (0.7)	0 (0)	0 (0)	1 (1.4)	2 (0.5)

*DTCSH* Debre Tabor Comprehensive Specialized Hospital, *NMWH* Nifas Mewcha Hospital, *ADZH* Addis Zemen Hospital, *MEYH* Mekane Eyesus Hospital

### Control of blood pressure level among hypertensive patients

Among participants, 41.4% had controlled blood pressure based on WHO target blood pressure recommendations whereas according to JNC-8 guideline recommendations, 52.2% of participants had controlled blood pressure.

### Factors affecting blood pressure control among hypertensive patients

A binary logistic regression showed that patients who didn’t have health insurance were less likely to have controlled BP compared to those who had health insurance (Crude Odds Ratio [COR] = 0.58 [0.37–0.91], Adjusted Odds Ratios [AOR] = 0.42[0.26–0.68]). Non-adherent patients to their antihypertensive medications were less likely to have controlled BP compared to those patients who were adherent to their medication (COR = 0.57[0.38–0.84], AOR = 0.52[0.34–0.80]). Concerning to regular aerobic exercise, patients who didn’t engage in regular aerobic exercise were less likely to have controlled BP compared to patients engaged in regular aerobic exercise (COR = 0.54[0.34–0.85], AOR = 0.53[0.32–0.88]). Non-compliance to WHO pharmacologic treatment of hypertension guideline in selecting antihypertensive drugs for the treatment of hypertension was less likely to achieve controlled BP compared to compliance to it (COR = 0.45[0.26–0.79], AOR = 0.47[0.26–0.85]). Regarding to the number of antihypertensive drugs, patients who were treated with monotherapy antihypertensive drug were more likely to have controlled BP in reference to patients who were treated with three or more antihypertensive drugs (COR = 3.93[1.55–9.98], AOR = 3.83[1.42–10.35]) (Table 5).

**Table 5:** Determination of variables association with blood pressure control

**Variables**	**Blood pressure**		**OR (95% CI)**	
	**Uncontrolled**	**Controlled**	**COR**	**AOR**
Sex				
Male	108	62	0.71(0.48–1.06)*	0.74(0.48–1.15)
Female	140	113	1	1
Marital status				
Single	20	7	1	1
Married	171	116	1.94 (0.79–4.73)*	1.56(0.62–3.97)
Divorced	15	17	3.24(1.07–9.79)*	2.06(0.63–6.75)
Widowed	42	35	2.38(0.90–6.29)*	1.85(0.65–5.26)
Monthly income				
< 1500 birr	67	39	0.69(0.43–1.14)*	0.71(0.40–1.25)
1500–2499 birr	83	54	0.78(0.49–1.22)	0.73(0.44–1.22)
≥ 2500 birr	98	82	1	1
Health insurance				
Yes	168	137	1	1
No	80	38	0.58(0.37–0.91)*	0.42(0.26–0.68)^+^
Comorbidity				
Yes	133	76	0.66(0.45–0.98)*	0.50(0.20–0.95)
No	115	99	1	1
Adherence status				
Adherent	80	80	1	1
Non-adherent	168	95	0.57(0.38–0.84)*	0.52(0.34–0.80)^+^
Aerobic exercise				
Yes	46	52	1	1
No	202	123	0.54(0.34–0.85)*	0.53(0.32–0.88)^+^
Hypertension specific MRCI				
Low MRCI	120	116	5.80(1.27–26.48)*	1.28(0.15–10.73)
Moderate MRCI	116	57	2.95(0.64–13.62)*	1.38(0.23–8.48)
High MRCI	12	2	1	1
Patient level MRCI				
Low total MRCI	35	54	3.39(1.96–5.87)*	2.04(1.07–3.91)^+^
Moderate total MRCI	112	75	1.47(0.93–2.32)*	1.17(0.72–1.91)
High total MRCI	101	46	1	1
Drug selection				
Appropriate	193	155	1	1
Inappropriate	55	20	0.45(0.26–0.79)*	0.47(0.26–0.85)^+^
Antihypertensive drugs				
One	113	111	3.93(1.55–9.98)*	3.83(1.42–10.35)^+^
Two	111	58	2.09(0.81–5.40)*	2.27(0.85–6.04)
Three or more	24	6	1	1
Drug-drug interaction				
Major	66	37	0.61(0.43–1.09)*	0.4(0.10–0.85)
Moderate	29	12	0.50(0.25–1.03)*	0.74(0.33–1.67)
No interaction	153	126	1	1

* and ^+^ stands for variables significant in univariate and multivariate analysis, respectively

*Abbreviations:AOR* Adjusted Odds Ratio, *COR* Crude Odds Ratio, *MRCI* Medication Regimen Complexity Index

## Discussion

In order to achieve target BP, prescribing appropriate medications at proper dose, frequency and duration, being adherent to medications and self-care practices are required [12, 23]. Assessing the present antihypertensive drug prescription practice with respect to the standard treatment guidelines will help to identify gaps ant ensure adequate BP control. In the current study, on average around 93% and 95% of prescriptions for hypertension treatment was in accordance with WHO and JNC-8 guideline, respectively. This finding was higher than results of the studies conducted in Kenya (82%) [6], Gondar University Hospital (66.85%) [11], and Jimma University Hospital (44.1%) [9]. The current study has found that prescription patterns affected the level of BP control. Inappropriate antihypertensive drug selection accounted for greater number of non-compliance to treatment guidelines compared to dose. Approximately, 82% and 87% of patients was treated with effective drugs as per WHO and JNC-8 guidelines, respectively. Drug selection criteria should take into account patients’ condition such as age, comorbidity and organ function status. WHO pharmacologic treatment for hypertension guideline recommends thiazide diuretics or calcium channel blockers for patients with over 65 years old and African descent, beta blockers for patients with ischemic heart disease, and ACEIs/ARBs for patients with severe proteinuria, diabetes mellitus or chronic kidney disease [2]. JNC-8 recommends thiazide diuretics or CCBs for patients from African descent including those with diabetes mellitus, ACEIs/ARBs for patients with severe proteinuria and chronic kidney disease [17]. The current study found that ineffective drug selection was negatively associated with BP control (AOR = 0.47[0.26–0.85]). This finding is supported by studies in United States (US) and Gondar University Hospital [11, 24].

Among monotherapy, hydrochlorothiazide was the most commonly prescribed antihypertensive drug (51.3%), followed by nifedipine (21%) and enalapril (14.3%). Regarding to hydrochlorothiazide monotherapy, the finding of our study is similar with other studies done in Hiwot Fana Specialized University Hospital [25], Gondar University Teaching Hospital [11], and Federal police Referral Hospital [23]. However, the second most commonly prescribed drug among monotherapy was nifedipine in our study which is not line with studies conducted in Ethiopia [11, 23, 25]. The most commonly prescribed among monotherapy was enalapril in a study conducted at Jimma University Hospital which is not in line with the current study and other studies in Ethiopia [11, 23, 25]. The discrepancy of prescription patterns of studies may be due to the difference in extent of adherence to treatment guideline recommendations, and the presence of comorbid illnesses. Monotherapy was associated with optimal blood pressure control compared to triple or more drug therapies in our study (AOR = 3.93[1.55–9.98]). This finding may be due to patients adherence to their medication is more in monotherapy as a result of less pill burden and less complex instructions than dual or more therapy and another study found that patients taking more than three antihypertensive medications were less likely to have controlled BP compared to those patients taking monotherapy [26]. In our study, low level of patient level medication regimen complexity was associated with optimal blood pressure control (AOR = 2.04[1.07–3.91]). This finding is supported by other studies which found that high level of medication regimen complexity was negatively associated with medication adherence and in turn, non-adherence to medications was positively associated with poor clinical outcomes [24, 27–29].

In our study, patients who didn’t follow regular aerobic exercise was less likely to achieve optimal blood pressure control compared to those patients who followed regular aerobic exercise (AOR = 0.53[0.32–0.88]). This result is in accordance with finding of a meta-analysis which concluded that aerobic exercise reduces blood pressure [30].

In the current study, not having health insurance was negatively associated with optimal blood pressure control compared to those who had health insurance (AOR = 0.42[0.26–0.68]). This finding is in line with the finding of the study conducted in United States [26].

This study has provided information regarding the prescription patterns of antihypertensive drugs with respect to the level of BP control. It is a multi-center study and it will help prescribers to focus on specific factors that affect BP control. But, it has some limitations. First, it is a cross sectional study. Second, data were collected by chart review and patient interview, so prescribers were not involved to give information about their attitude towards compliance to treatment guideline and factors affecting it. Hence, a prospective study having discussion with prescribers is needed to ascertain factors affecting adherence to treatment guidelines and blood pressure control. In addition, inappropriate prescriptions might be overestimated by our study as pharmacists might have responded for some inappropriate prescriptions after prescription.

## Conclusion

The finding of the present study showed that on average prescribers adherence to WHO guideline was not 100%. Inappropriate drug selection was the main contributing factor for violation of the guideline recommendation. High drug-drug interactions (one-third of participants) were found. Patient level low MRCI and monotherapy were positively associated with BP control whereas, non-adherence, inappropriate drug selection, having no health insurance, and didn’t follow regular aerobic exercise were negatively associated with BP control. Clinicians should adhere to treatment guidelines and focus on modifiable factors to improve BP control. Ethiopian heath minster and Health bureau shall identify the gaps why prescribers do not adhere to guidelines and design interventions.

## Data Availability

Data analyzed and used for this manuscript are available within the manuscript.
